# Supplementary material for: Photo-induced halide redistribution in organic–inorganic perovskite films
Source: Nat Commun. 2016 May 24;7:11683. doi: 10.1038/ncomms11683 (PMC4890321; doi:10.1038/ncomms11683)
Supplement: Supplementary Information — Supplementary Figures 1-16, Supplementary Notes 1-13, Supplementary Methods and Supplementary References. [file ncomms11683-s1.pdf]

Supplementary Information

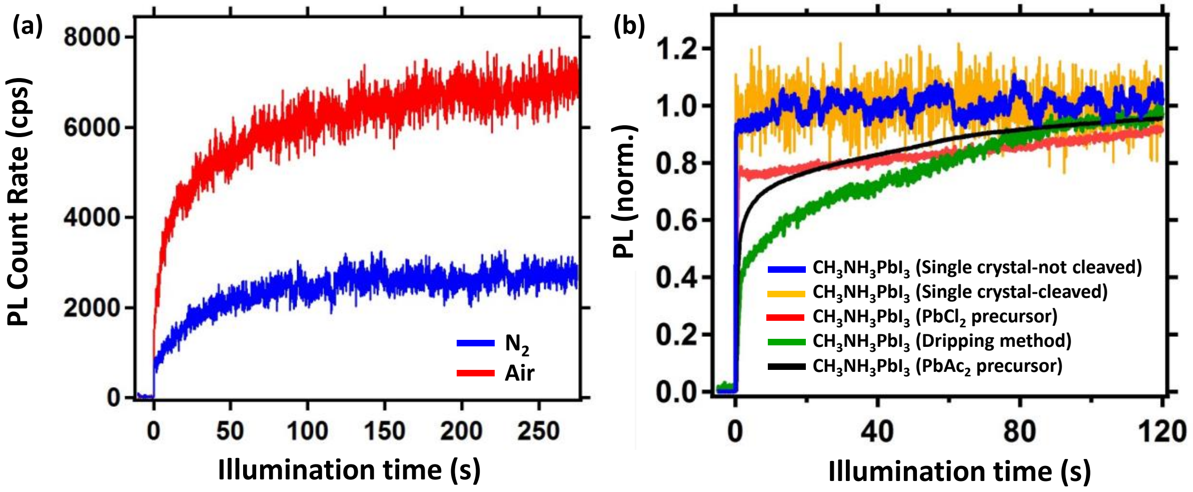

Supplementary Figure 1. **Photoluminescence (PL) rise behaviour in different atmospheres and for different processing conditions.** (a) PL rises over time under illumination (532 nm CW, 60 mW cm^{-2}) of a sample first measured in air and then in nitrogen, demonstrating that the atmosphere can affect the magnitude of PL enhancement but the rise is present in both conditions[1-3]. (b) PL rises over time under illumination of several perovskite samples prepared with various processing techniques and measured in nitrogen, showing that the rises are generally observed in polycrystalline films, but not in single crystals. The films[4,5] and single crystals[6] were processed as described in the Supplementary Methods. The HPA/acetate[7] films were used throughout the remainder of the work. The polycrystalline films and single crystals were excited with a 532 nm CW laser with intensities of 160 mW cm^{-2} and 300 mW cm^{-2}, respectively.

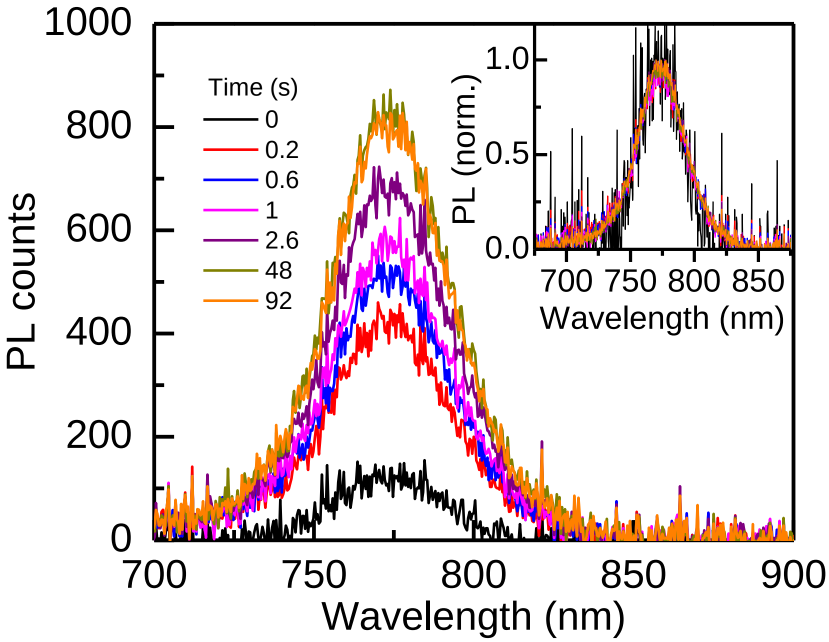

Supplementary Figure 2. Monitoring photoluminescence (PL) spectra in time. PL spectra from a $CH_3NH_3PbI_3$ film over time under illumination with a continuous wave (CW) laser at a wavelength of 532 nm, with an intensity of ~60 mW cm^{-2}, producing photo-excitation densities comparable to 1-sun AM 1.5 illumination. The emission was collected using a fiber-coupled Ocean Optics Mayapro spectrometer with integration times of 200 ms. The inset shows the normalized spectra, indicating that the PL spectral shape and position do not significantly change over time.

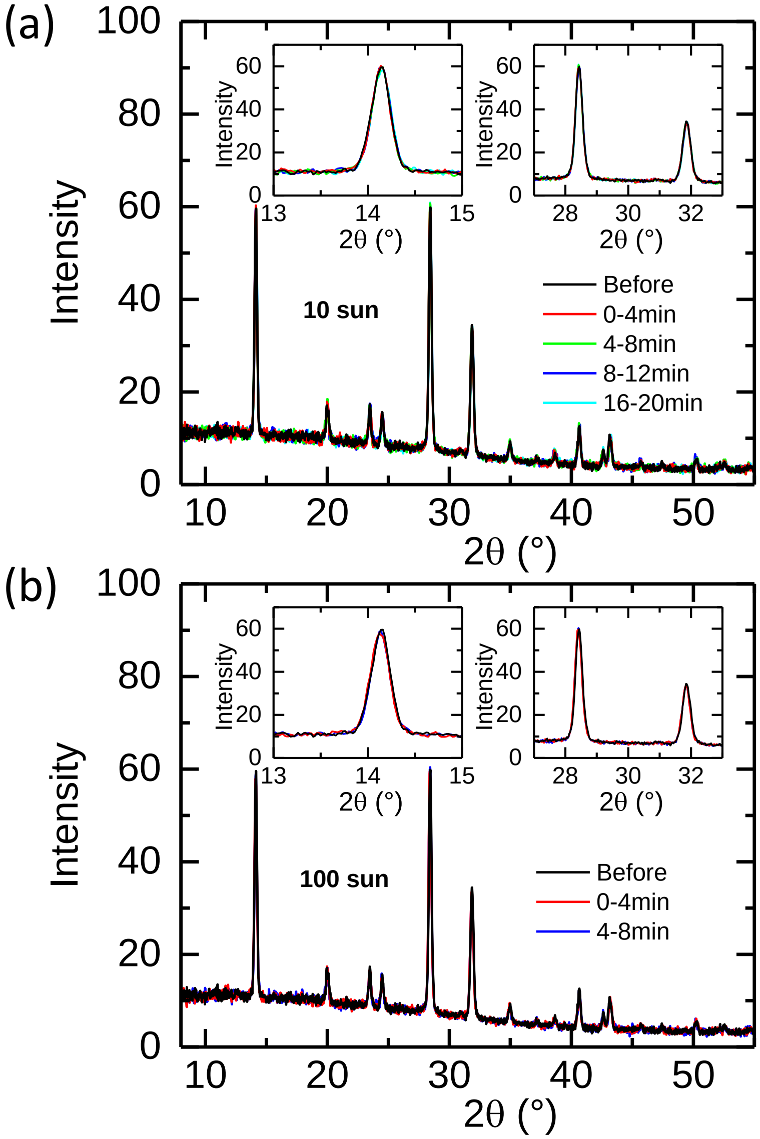

Supplementary Figure 3. Monitoring structural changes of films under illumination. X-Ray Diffraction (XRD) patterns of a $CH_3NH_3PbI_3$ film acquired (4 minute integration time) before illumination and during various windows while under constant illumination with a laser at a wavelength of 532 nm, with an intensity of (a) ~600 mW cm^{-2} (~10 sun equivalent, total photon dose of ~0.7 kJ cm^{-2}) and (b) ~6000 mW cm^{-2} (~100 sun equivalent, total photon dose of ~3 kJ cm^{-2}). No significant changes were observed.

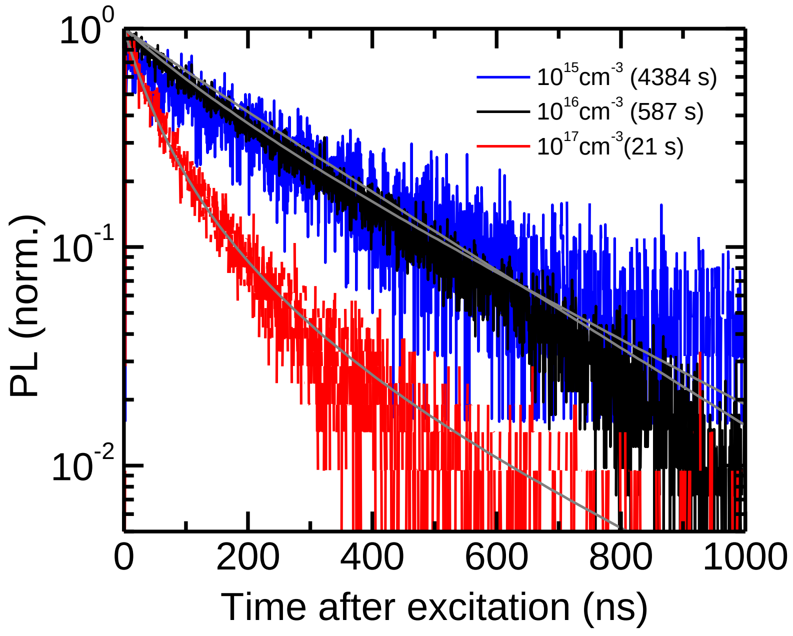

Supplementary Figure 4. **Intensity dependent bulk time-resolved photoluminescence (PL).** Stabilized PL decays detected at 780 nm from the $CH_3NH_3PbI_3$ sample following pulsed excitation (507 nm, 1 MHz repetition rate) with different initial photoexcitation densities *N*(0). The bracketed numbers indicate the times (in seconds) taken to reach stabilized emission. Solid gray lines are fits from the model.

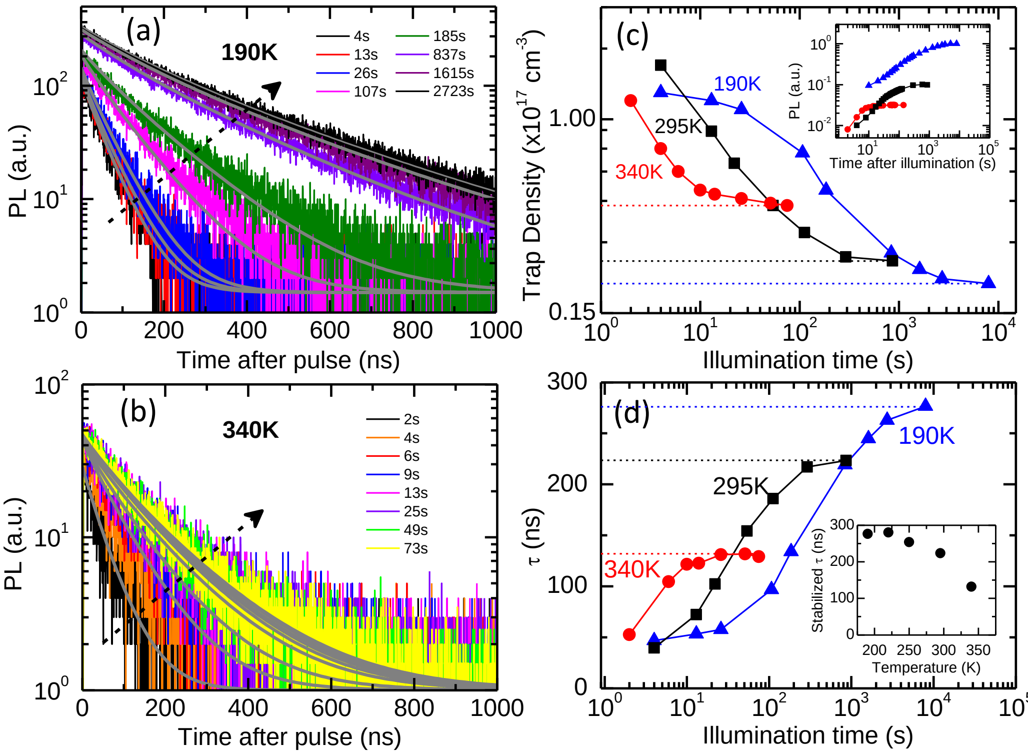

Supplementary Figure 5. **Temperature-dependent PL changes.** A selection of time-resolved PL decays from a thin $CH_3NH_3PbI_3$ film measured over time under illumination at low temperature (190 K) (a) and high temperature (340 K) (b). The stated times in the legend are time stamps at the end of the integration window for each curve. The sample was photoexcited with pulsed excitation (507 nm, 1 MHz repetition rate, 117 ps pulse length and 0.3 μJ cm^{-2} per pulse, which creates a photo-excited species density of ~10^{16} cm^{-3}) and the emission was detected at 780 nm. The gray lines represent fits to the data from the model described in the text. (c) Trap densities extracted from the fits to the data in (a), (b) and Figure 1a (main text). The dashed lines represent the stabilized trap densities. *Inset*: The unnormalized integrated PL over time under initial illumination determined from integrating acquired PL decays at each temperature. (d) Monomolecular lifetimes τ extracted from mono-exponential fits to the PL data over time under illumination at each temperature. *Inset*: The stabilized lifetimes as a function of temperature.

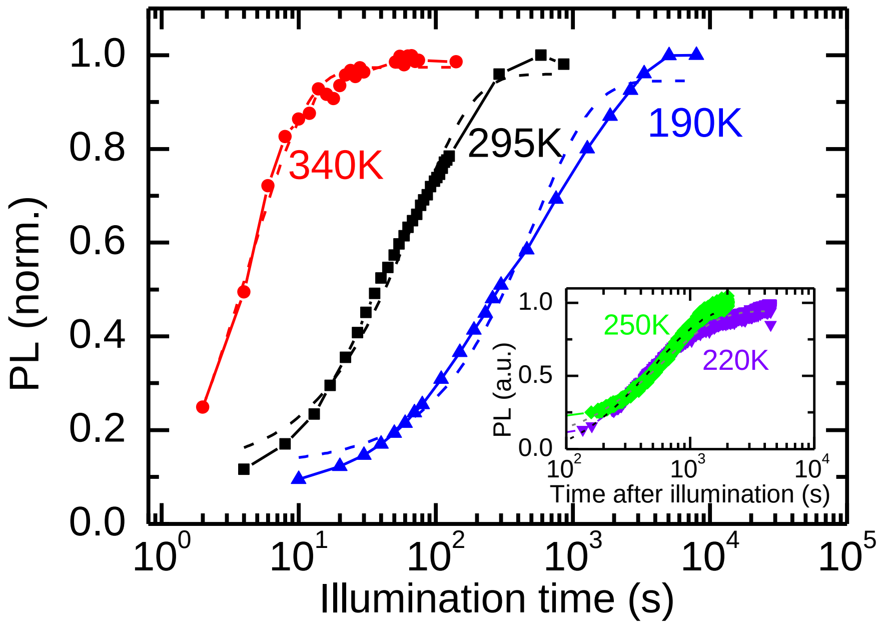

Supplementary Figure 6. Temperature-dependent PL rises. The normalized integrated PL over time under initial illumination determined from integrating acquired PL decays at each temperature, where the time corresponds to the end of the integration window. *Inset*: The integrated PL data over time at 250 K and 220 K. The dashed lines are single exponential fits to the rises to extract approximate time constants τ for the Arrhenius fits in Figure 1c inset (main text).

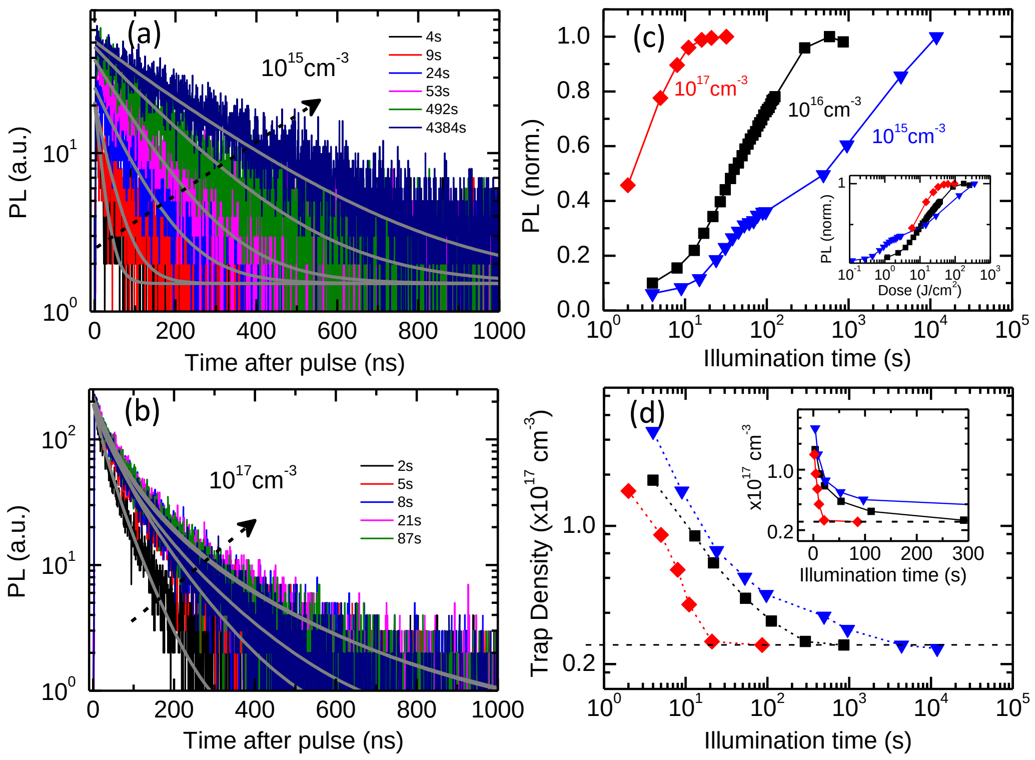

Supplementary Figure 7. **Intensity-dependent PL changes under illumination**. A selection of time-resolved PL decays from a thin $CH_3NH_3PbI_3$ film measured over time under illumination at (a) low excitation fluence (0.03 μJ cm^{-2} per pulse, N_0 ~10^{15}cm^{-3}) and (b) high excitation fluence (3 μJ cm^{-2} per pulse, N_0 ~10^{17}cm^{-3}). The samples were excited at 507 nm, 1 MHz repetition rate, 117 ps pulse length at room temperature, and the PL detected at 780 nm. The stated times in the legend are time stamps at the end of the integration window for each curve. The gray lines represent fits to the data from the model described in Supplementary Note 3. (c) The normalized integrated PL over time under initial illumination determined from integrating acquired PL decays at each fluence. Inset: The same data plotted with total light-soaking dose as the x-axis. (d) Trap densities extracted from the fits to the data in (a), (b) and Figure 1a (main text). The dashed lines represent the stabilized trap densities, as represented by the trap densities extracted from the fits in Supplementary Fig. 4. *Inset*: The same data but on a linear time scale over the first 300s.

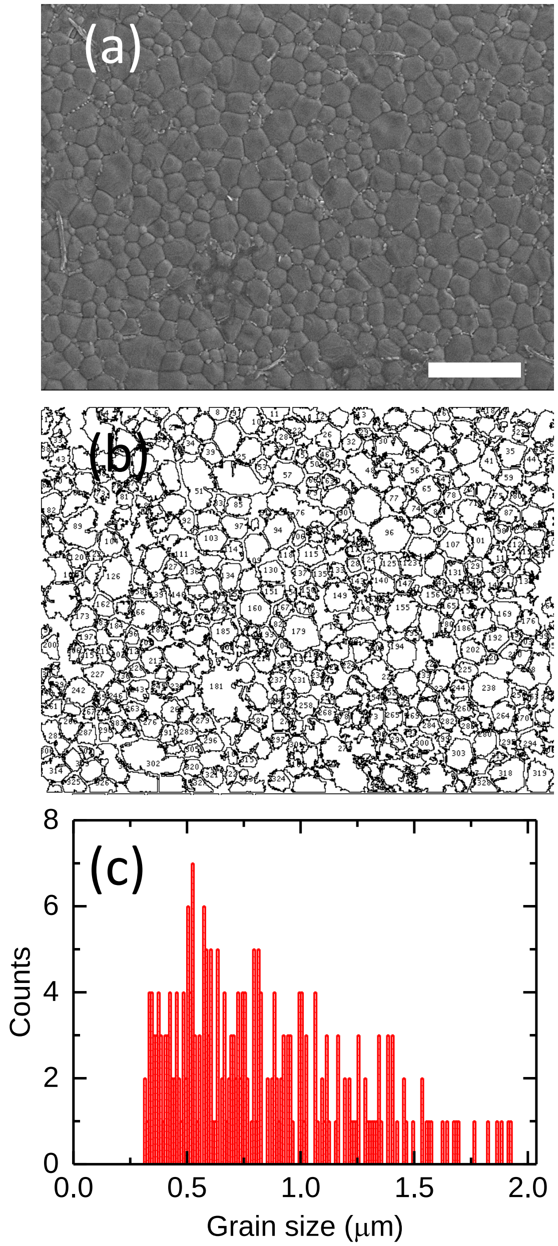

Supplementary Figure 8. Grain size analysis via scanning electron microscopy (SEM) (a) SEM image of the perovskite films, scale bar is 5 μm. (b) Processed SEM image using ImageJ software employing a built-in Gaussian Blue filter, Enhance Contrast and Find Edges operations with grains smaller than 100 nm filtered out to avoid incorrect assignments of grains. (c) Histogram of grain sizes from (b) calculated from the measured grain surface areas.

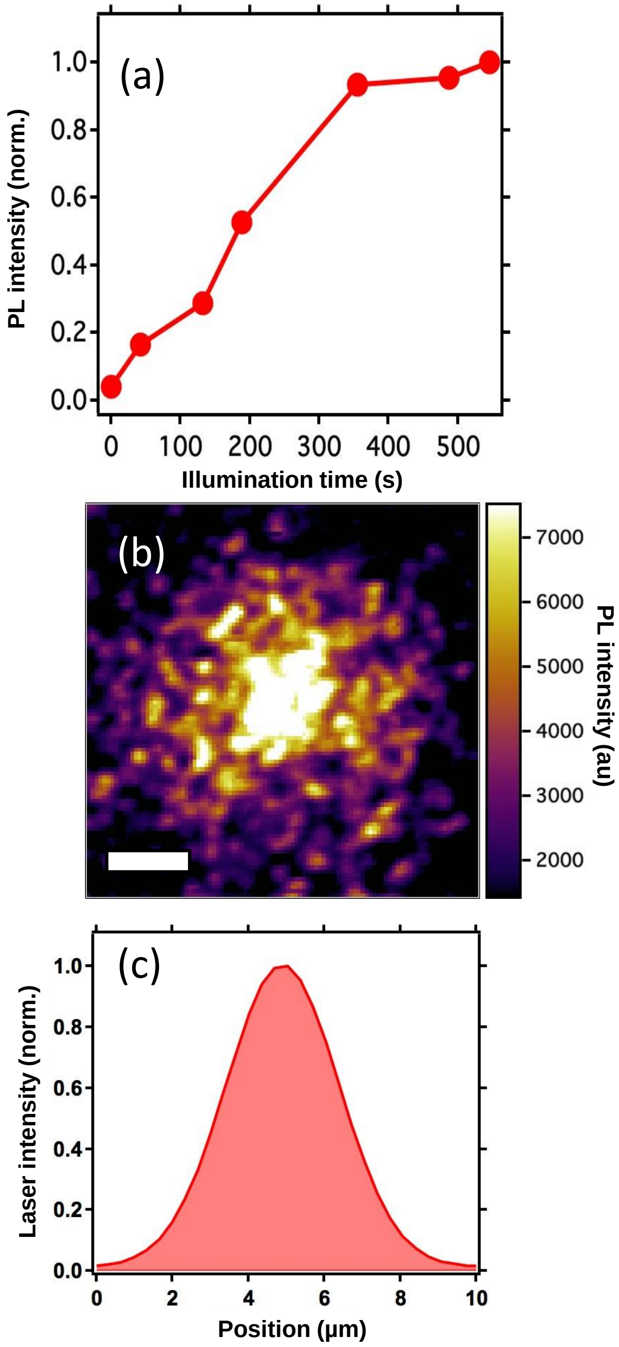

Supplementary Figure 9. PL enhancement compared to laser profile (a) PL rise over time under illumination (470 nm, 40 MHz repetition rate, 0.1 μJ cm^{-2} per pulse). (b) PL map of a wider area showing that the brightening closely follows the laser excitation spatial profile in (c), scale bar is 2 μm.

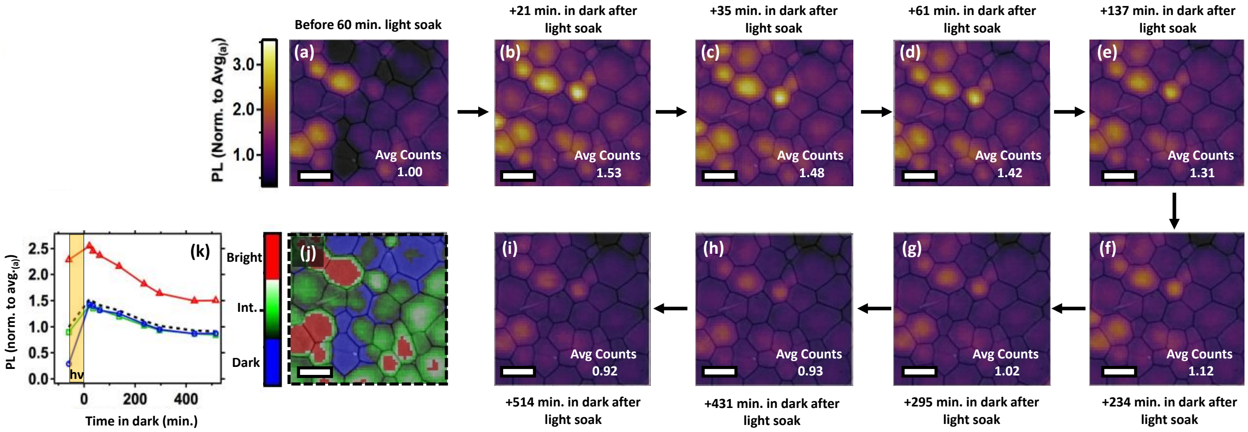

Supplementary Figure 10. Local grain emission relaxation. Fluorescence images under pulsed excitation (470 nm, 40 MHz repetition rate, 350 ps pulse length and 0.03 μJ cm^{-2} per pulse) in nitrogen with semitransparent SEM images overlaid (a) before light soaking, and after exposing the entire film to simulated sunlight (AM 1.5, 100 mW cm^{-2}) for 60 minutes and leaving in the dark for (b) 21, (c) 35, (d) 61, (e) 137, (f) 234, (g) 295, (h) 431, and (i) 514 minutes, scale bar is 1 μm. (j) Three-colour scale image showing the regions classified as dark (blue < -σ from avg. in (a)), intermediate (-σ from avg. ≤ green ≤ +σ from avg.) and bright (red > +σ from avg.). (k) Local PL enhancements for a dark (blue), intermediate (green), and bright regions as indicated in (j), where the time under illumination is highlighted by the yellow shaded region for t < 0, and the times t ≥ 21 show the local PL relaxation dynamics over time left in the dark. The dotted black line is the PL relaxation averaged across the whole fluorescence image.

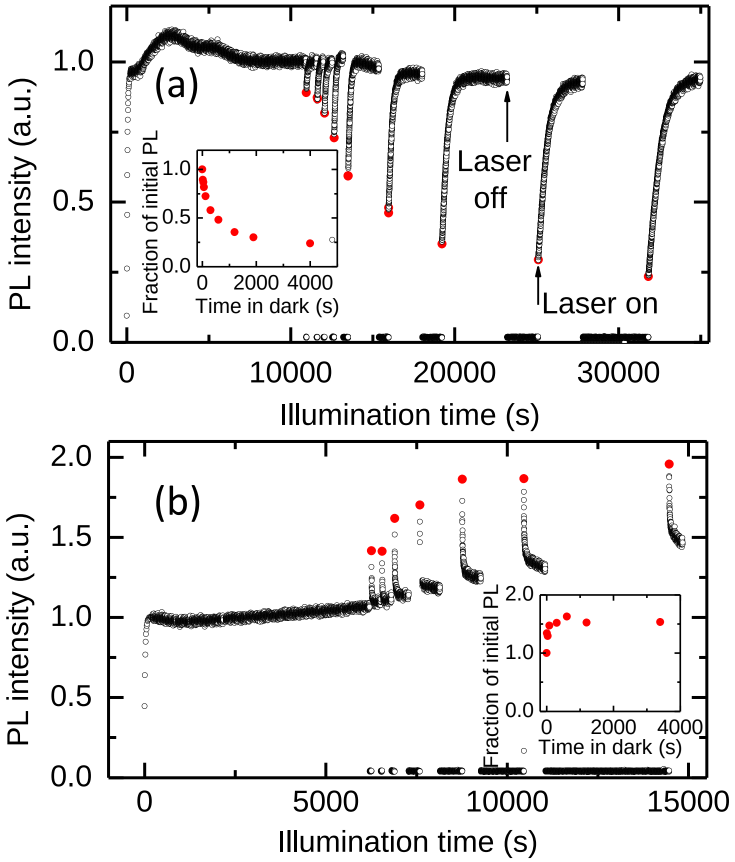

Supplementary Figure 11. Bulk film relaxation in the dark. PL intensity after leaving films in the dark (no illumination) for varying times. In each case, the PL is first allowed to reach a stabilized emission under illumination, then the laser switched off for a fixed length of time and then switched back on, with the PL continually monitored. The red closed circles represent the value of the PL intensity immediately after switching on the laser. The inset shows the PL intensity, relative to the stabilized emission before switching the laser off, for different times in the dark. (a) and (b) are two different scenarios observed in identical (duplicate) films measured under identical conditions. The samples were photoexcited with pulsed excitation (w ~ 17 μm, 507 nm, 1 MHz repetition rate, 117 ps pulse length and 0.3 μJ cm^{-2} per pulse, which creates a photo-excited species density of ~$10^{16} cm^{-3}$) and the emission at 780 nm is time-integrated.

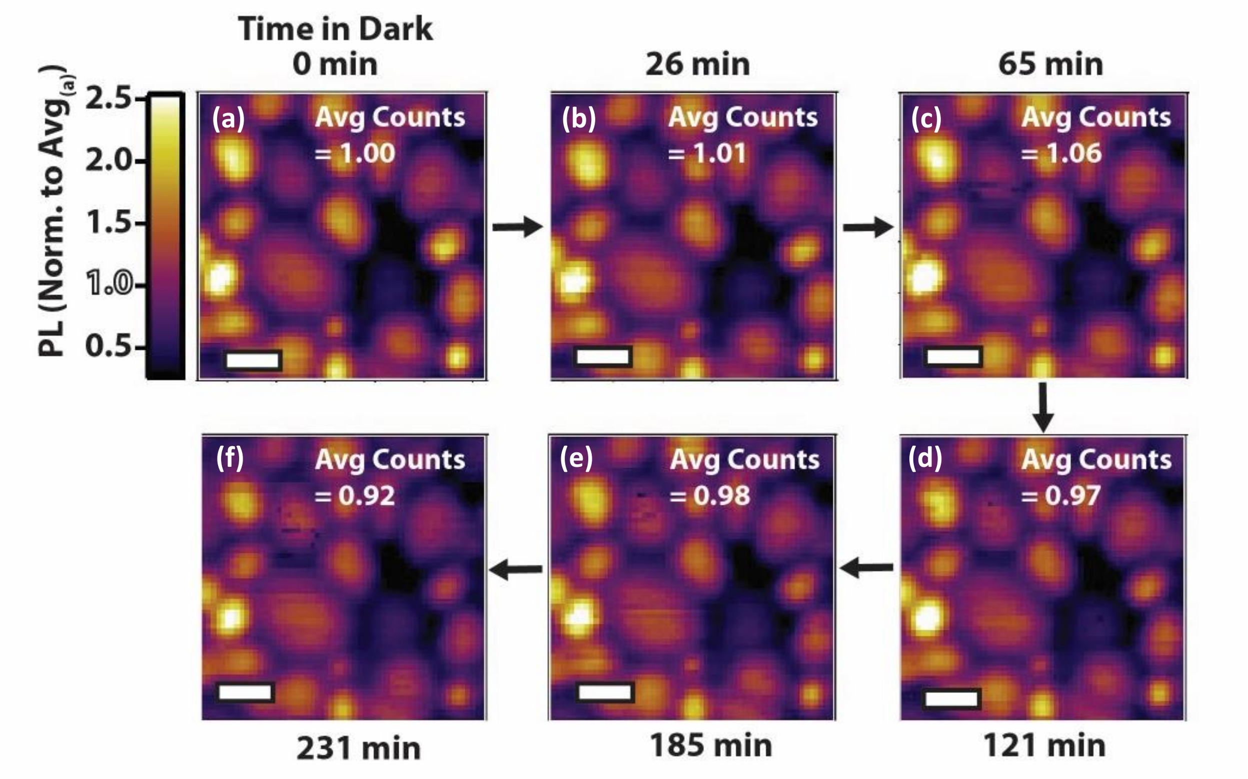

Supplementary Figure 12. Local grain emission and relaxation without light soaking. Fluorescence images under pulsed excitation (470 nm, 40 MHz repetition rate, 350 ps pulse length and 0.03 μJ cm^{-2} per pulse) in nitrogen taken at (a) 0 (b) 26, (c) 65, (d) 121, (e) 185, and (f) 231 minutes showing that a long light soak is required to redistribute the emission intensities, scale bar is 1 μm.

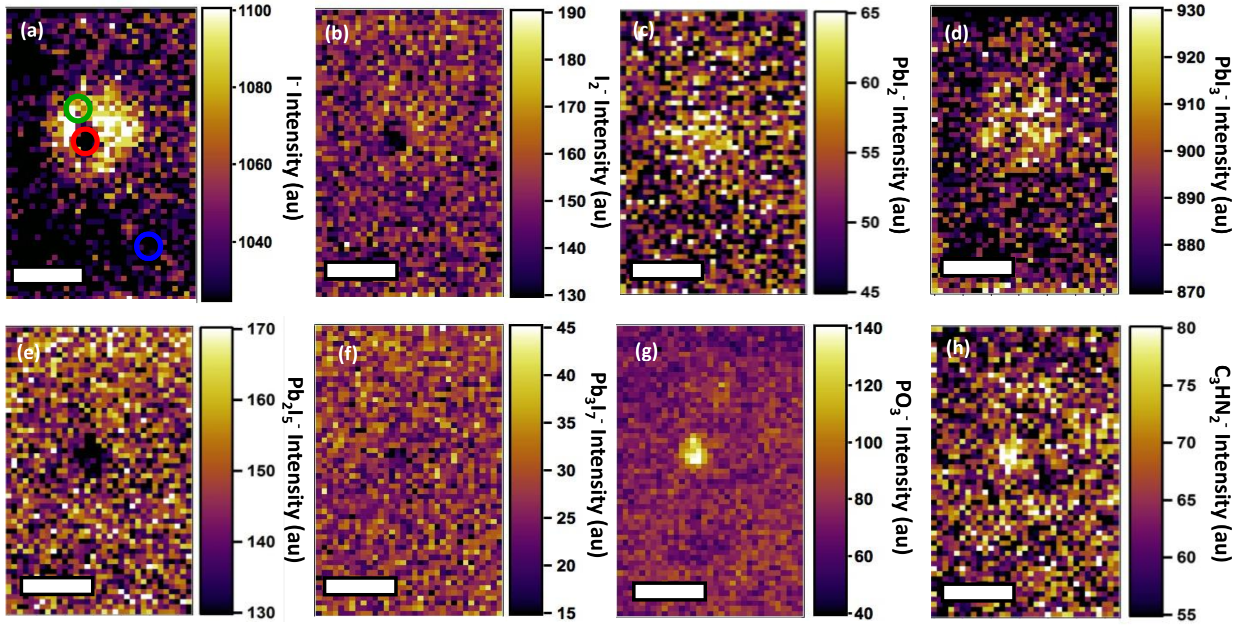

Supplementary Figure 13. **ToF-SIMS intensity images**. (a) ToF-SIMS image of the I^-, (b) I_2^-, (c) PbI_2^-, (d) PbI_3^-, (e) $Pb_2I_5^-$, (f) $Pb_3I_7^-$, (g) PO_3^{3-}, and (h) $C_3HN_2^-$ fragments and their distribution summed through several layers of the entire film, scale bar is 10 µm. The red spot shows where the sample has been light soaked, the green spot an immediately adjacent spot, and the blue spot represents a background region that has not been illuminated.

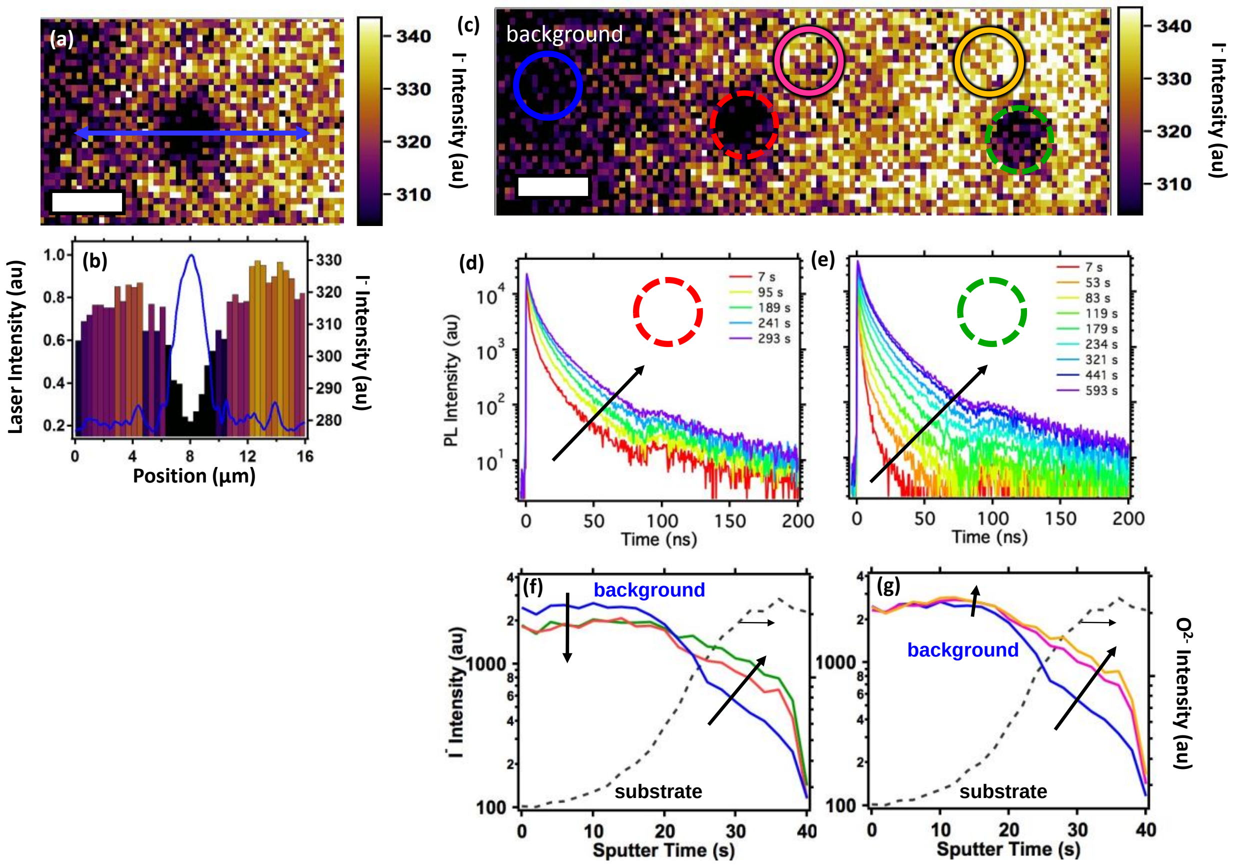

Supplementary Figure 14. **ToF-SIMS and time-resolved PL measurements of $CH_3NH_3PbI_3$ films.** (a) ToF-SIMS image of region exposed to 1.2 kJ cm^{-2} in the main text (Figure 5a) and the measured laser profile (b) across the line scan in (a), scale bar is 5 μm. (c) ToF-SIMS image of the iodide distribution (the image has been adjusted to show maximum contrast) indicating five regions of interest, where the blue circle represents a background region without any illumination, the red region has been exposed to 1.2 kJ cm^{-2}, and the green region to 2.4 kJ cm^{-2}, and the pink and gold circles are adjacent regions. The corresponding PL decays over time are shown for the (d) red region and (e) green region from (c). (f and g) Raw intensity depth profiles of the various regions of interest with the oxygen intensity (from substrate) indicated by dashed black lines. All selected regions contained the same number of pixels to allow comparisons.

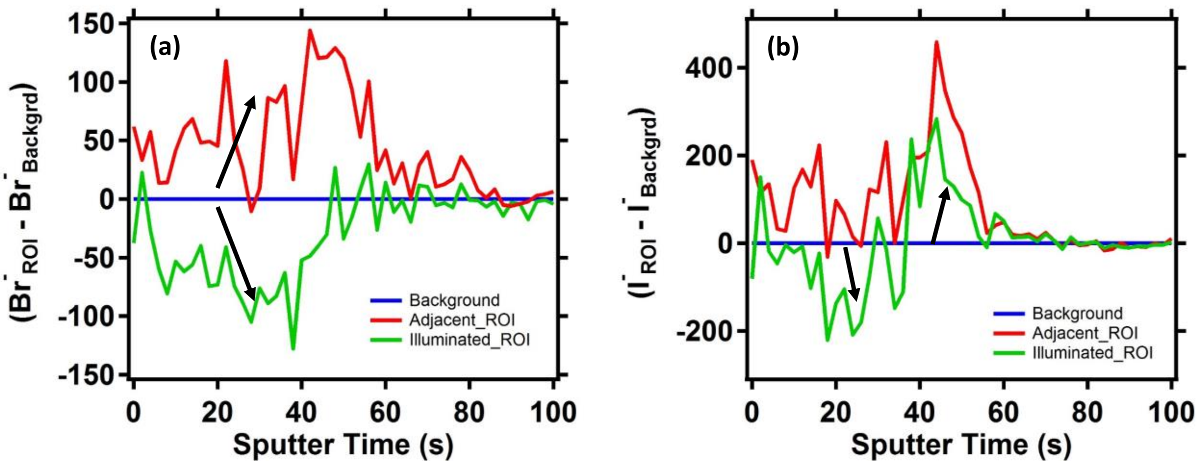

Supplementary Figure 15. ToF-SIMS depth profile data of an illuminated $CH_3NH_3Pb(I_{1-x}Br_x)_3$ film (x=0.4). (a) ToF-SIMS depth profile data of the bromide content relative to the background ($Br^-_{ROI} - Br^-_{background}$) for an illuminated (green) and adjacent (red) region. (b) Depth profile data of the iodide content relative to the background ($I^-_{ROI} - I^-_{background}$) for an illuminated and adjacent region.

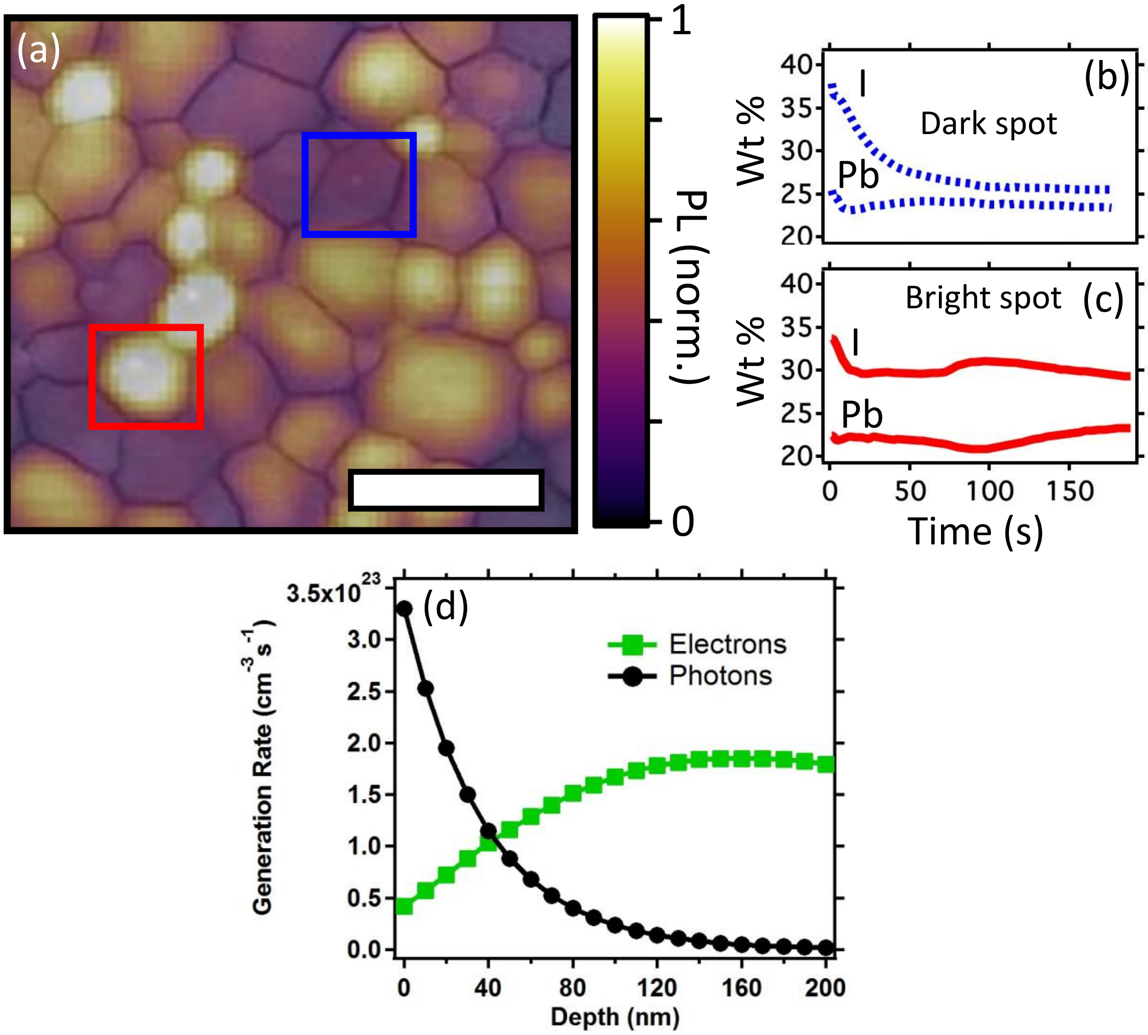

Supplementary Figure 16. **Monitoring iodide content over time via energy dispersive X-ray spectroscopy (EDS).** (a) Composite image of semitransparent SEM micrograph of a perovskite film overlaid on a PL image, scale bar is 2 μm. EDS measurements following lead and iodide weight fractions over time under illumination with a 10 keV electron beam at a (b) dark emission spot (blue) and (c) bright emission spot (blue). (d) Calculated electron-hole generation rates under the optical or electron beam excitation used for PL and EDS measurements, respectively.

Supplementary Note 1. Effect of Atmosphere and Film Preparation on Photoluminescence Transients

Unlike some recent reports[1,2], we do not believe that our observations can be attributed solely to atmospheric effects because we see the transient rise behavior when measuring under vacuum, in nitrogen or in air (Supplementary Fig. 1), although we note that atmospheric conditions such as humidity and oxygen can also influence the film and emission properties, particularly over long time scales (such as storage under these conditions) or when processing under uncontrolled atmospheric conditions[3,8,9].

Supplementary Note 2. Monitoring Bulk Film Properties Over Time Under Illumination

We monitor in time the bulk photoluminescence (PL) spectra (Supplementary Fig. 2) and any structural changes via X-Ray Diffraction (XRD) (Supplementary Fig. 3) over time under illumination.

Supplementary Note 3. Model to Describe Recombination Kinetics

The model to describe the recombination kinetics in the presence of subgap states is derived in detail in our recent work[10], but we summarize the salient points here with some additional modifications. Here, we consider only electronic (subgap) traps, though the same formalism holds for hole traps.

Under pulsed illumination, an optical pulse excitation generates an electron-hole pair density of $N(0)$. There are N_T electron traps, n_T of which are filled leaving n_T free "photo-doped" holes in the valence band such that the total concentration of holes at each point in time is $n_h = n_e + n_T$, where n_e is the concentration of free photogenerated electrons. The total concentration of photo-

generated species $N = n_e + n_x$ is thus comprised of concentrations of free electrons and excitons n_x, but excludes trapped electrons and corresponding photo-doped holes. We note that we include excitons here for completeness but due to the low exciton binding energy in these materials, their presence does not significantly affect the results.

In Supplementary Fig. 4 we show transient PL decays at room temperature over a range of pulse fluences, where the sample was first illuminated at each fluence until a steady transient emission was achieved. The shape of the decays is nearly mono-exponential at relatively low excitation fluence, but deviates significantly from mono-exponential at higher fluence. In the steady state, there are many holes already present in the system as 'background' or 'photo-doped' charges because some of the electronic traps are filled. When the sample is photo-excited with a low fluence, the concentration of photo-induced electrons is much lower than the total concentration of free holes (since $n_h(t) = n_e(t) + n_T(t)$, $n_e \ll n_T$), and the recombination of electrons is almost mono-molecular since the additional photo-excited charge does not noticeably change the concentration of holes. If the excitation fluence is high enough, such that the concentrations of photo-excited electrons and holes from the laser pulse become comparable to the photo-doped hole density ($n_e(0) > n_T \sim N_T$), then the electron-hole recombination is bimolecular resulting in a power law decay until the free electron concentration drops below the photo-doped hole density, after which the decay again becomes pseudo mono-exponential.

A generic kinetic model, accounting for exciton formation, dissociation into free charges and trapping of free electrons, is given by the rate equations

$$\frac{dn_e}{dt} = \frac{I}{d} + R_d n_x - R_f n_e n_h - R_{eh0} n_e n_h - R_{eh1} n_e n_h \left(N_T - n_T\right) - R_{pop} n_e \left(N_T - n_T\right) \quad (1)$$

$$\frac{dn_x}{dt} = R_f n_e n_h - R_d n_x - R_x n_x \quad (2)$$

$$\frac{dn_T}{dt} = R_{pop}\left(N_T - n_T\right)\cdot n_e - R_{dep}\cdot(n_T^2 + n_T\cdot n_e) \quad (3)$$

The parameters R_f, R_d, and R_x are the rates of exciton formation, dissociation and decay, respectively. R_{eh0} and R_{eh1} describe electron recombination through band-to-band and trap-assisted (Shockley-Read-Hall), respectively, R_{pop} and R_{dep} determine trap population and depopulation by electrons, respectively, where depopulation is exclusively by recombination with either free (photo-generated) or photo-doped holes and we assume the traps are deep enough to inhibit thermal detrapping to the conduction band; I is the excitation intensity (I = 0 for pulsed excitation), d is the film thickness, and N_T is the total trap concentration.

Before solving this model we make two further simplifications: 1) the accumulation of charges in traps and their depopulation take place on a time scale of milliseconds to seconds, suggesting that the trap concentration n_T can be taken constant when we consider PL decays on time scales of microseconds. Therefore on the time scales for the evolution of n_e or n_x we set $n_T = Const$, 2) Due to the very fast exciton formation and dissociation, the free carriers and excitons can be assumed to be in thermal equilibrium throughout the entire PL decay.

We can then analytically solve (1)-(3). For consistency with our previous work, we ignore the term involving R_{eh1}, which also effectively takes into account trap-assisted electron recombination in a simplified way avoiding a highly nonlinear equation for n_T. This gives the following expression for the normalized PL:

$$\frac{I_{eh}(t)}{I_{eh}(0)} = \frac{I_{ex}(t)}{I_{ex}(0)} = \frac{n_e(t)\cdot n_h(t)}{n_e(0)\cdot n_h(0)}$$
$$= \frac{1}{(n_T + Ax_0)\cdot Ax_0}\cdot\left(n_T + \frac{AC_1x_0\exp[-\gamma\cdot t]}{C_1 + x_0(1-\exp[-\gamma\cdot t])}\right)\cdot\frac{AC_1x_0\exp[-\gamma\cdot t]}{C_1 + x_0(1-\exp[-\gamma\cdot t])} \quad (4)$$

where $A = \frac{v_x}{v_h v_e}\exp[-E_b / k_B T]$ and $v_i = \lambda_i^3$, λ_i is the thermal wavelength of the species *i* (we set E_b = 5 meV [11]), $x_0 = N(t=0)/(A+n_T)$, $\gamma_0 = AR_{eh} + R_x$ is the total rate of electronic decay not involving traps, and

$$\gamma = \frac{n_T\cdot\gamma_0}{A+n_T} + \frac{AR_{pop}\cdot(N_T - n_T)}{A+n_T}, C_1 = \frac{n_T}{A} + \frac{R_{pop}\cdot(N_T - n_T)}{\gamma_0}$$

$$n_T = -\frac{1}{2}\alpha + \frac{1}{2}\sqrt{\alpha^2 + 4\beta N_T}, \alpha = \frac{\left(A + \frac{R_{pop}}{R_{dep}}(A - N_T)\right)}{\left(1 + \frac{1}{K} + \frac{R_{pop}}{R_{dep}}\right)}, \beta = \frac{R_{pop}\cdot A}{R_{dep}\cdot\left(1 + \frac{1}{K} + \frac{R_{pop}}{R_{dep}}\right)} \quad (5)$$

with $K = \frac{1}{\gamma_0 t_0}\ln\left[1 + \frac{AN(0)}{N_T\cdot(A + N_T)}\right]$.

We set the following parameters to be the same as our previous work: R_{pop} = 2x10^{-10}cm^{3}s^{-1}, R_{dep} = 8x10^{-12}cm^{3}s^{-1}, and fix γ_0 =1.6x10^{7}s^{-1}. This leaves the only fitting parameter to be the trap density N_T. We globally fit the data across three orders of magnitude in Supplementary Fig. 4 (gray lines) to extract a common stabilized trap density of N_T = 2.5 x 10^{16} cm^{-3} at 300 K. The PL decays over time under illumination are then fit with the same parameters at all temperatures but by varying N_T. We note that the solution including the R_{eh1} term will be presented in a

forthcoming publication, but excluding it here is acceptable given we are primarily concerned with the relative changes in trap density under illumination.

Supplementary Note 4. Relation Between Photoluminescence Intensity and Trap Density

To illustrate the relationship between average PL intensity and the trap density, we rewrite the equation (1) for electronic density in a more generic and rigorous form (as we are not interested here in obtaining an analytical expression for n_T):

$$\frac{dn_e}{dt} = -\gamma \cdot n_e \cdot \left(n_e + n_T\right), \quad (6)$$

with the effective recombination rate containing the contributions from both the direct band-to-band electron-hole recombination and the trap-assisted (Shockley-Reed-Hall) recombination according to

$$\gamma = \gamma_0 + \beta \cdot \left(N_T - n_T\right), \quad (7)$$

where we used the notations $\gamma_0 = R_{eh0} + R_x / A$ for direct band-to-band recombination (radiative and non-radiative), and $\beta = R_{eh1}$ for trap-assisted recombination constant possibly dependent on temperature. Both N_T and n_T are constants on the time scale of PL decay. With this in mind the solution to Eq. (6) is found in the form

$$n_e = \frac{n_T \cdot n_e\left(0\right) \cdot \exp\left(-\gamma n_T \cdot t\right)}{n_T + n_e\left(0\right) \cdot \left(1 - \exp\left(-\gamma n_T \cdot t\right)\right)} \quad (8)$$

Assuming low exciton concentration relative to $n_e\left(0\right)$ the latter can be taken as the concentration of all absorbed photons from the excitation pulse and is typically in the range $10^{15} - 10^{17}$ cm^{-3}. The

expression above has a clear physical meaning. At low excitation fluence $n_e(0) << n_T$ and $n_e(t) \approx n_e(0) \cdot \exp(-\gamma n_T \cdot t)$, i.e. the decay of photo-excited electrons is mono-exponential due to monomolecular-type recombination. In the extreme opposite case $n_e(0) >> n_T$ and short times $\gamma \cdot n_T << 1$ the decay is hyperbolic $n_e(t) \approx n_e(0) \cdot (1 + n_e(0) \cdot \gamma \cdot t)^{-1}$ due to nearly-bimolecular recombination. At longer times the recombination becomes monomolecular again with the corresponding decay regime of the photoexcited electrons.

The time-in integrated PL (over the pulse period of duration t_0) calculated using Eq. (8) is

$$\langle I_{PL} \rangle \propto \langle n_e n_h \rangle =$$
$$\frac{\gamma_{rad}}{t_0} \int_0^{t_0} \left(\frac{n_T \cdot n_e(0) \cdot \exp(-\gamma n_T \cdot t)}{n_T + n_e(0) \cdot (1 - \exp(-\gamma n_T \cdot t))} \right) \cdot \left(\frac{n_T \cdot n_e(0) \cdot \exp(-\gamma n_T \cdot t)}{n_T + n_e(0) \cdot (1 - \exp(-\gamma n_T \cdot t))} + n_T \right) dt \approx \frac{\gamma_{rad} n_e(0)}{\gamma t_0}, \quad (9)$$
$$\gamma n_T t_0 >> 1$$

Substituting here Eq. (7) we obtain

$$\langle I_{PL} \rangle \approx \frac{\gamma_{rad} n_e(0)}{\gamma t_0} = \frac{\gamma_{rad} n_e(0)}{(\gamma_0 + \beta \cdot (N_T - n_T)) t_0} \quad (10)$$

If we compare the samples under constant pulse fluence then most likely $n_T \approx C \cdot N_T$, where the constant $C<1$, and we may write

$$\langle I_{PL} \rangle \approx \frac{\gamma_{rad} n_e(0)}{(\gamma_0 + \beta \cdot N_T \cdot (1 - C)) t_0}. \quad (11)$$

Finally, the second term in the denominator will dominate over the first because the trap densities N_T are large, such that:

$$\langle I_{PL} \rangle \propto \frac{1}{N_T} \tag{12}$$

The inverse relationship given by Eq. (12) is shown in Figure 1b of the main text.

Supplementary Note 5. Temperature-Dependence of Photoluminescence Rises

We show the time-resolved PL decays measured *in vacuo* at low temperature (190 K) in Supplementary Fig. 5a and at high temperature (340 K) in Supplementary Fig. 5b. We note here that the temperature range is chosen to avoid the phase transition in the perovskite at ~160K from a tetragonal to orthorhombic phase, and to avoid high temperatures (>350 K) where degradation will likely occur[12,13]. We fit the time-resolved PL decays measured over time under illumination for each temperature, and the extracted trap densities as a function of time under illumination are shown in Supplementary Fig. 5c. After reaching stabilized emission levels, the trap densities reach temperature-dependent values, with lower trap densities at low temperature ($N_T \sim 2 \times 10^{16}$ cm^{-3} at 190 K) compared to those at high temperature ($N_T \sim 2.5 \times 10^{16}$ cm^{-3} and 4.3×10^{16} cm^{-3} at 295 K and 340 K, respectively). We note that this temperature-dependence of the trap density is consistent with the longer stabilized monomolecular lifetimes (Supplementary Fig. 5d inset) and higher PL intensities (Supplementary Fig. 5c inset) at lower temperature (τ = 280 ns at 190 K) than at high temperature (τ = 132 ns at 340 K). These observations are also consistent with our earlier report where we showed that the PLQE approaches 100% at 190 K[10].

Supplementary Note 6. Arrhenius Fits

We fit single exponential functions to the rise in PL over time (Supplementary Fig. 6) to extract a time constant τ for each temperature. We then plot the rate constants $k=1/\tau$ versus 1/T and fit the data to the Arrhenius relation $k = A\exp(-E_a/RT)$ (where A is a prefactor, R is the ideal gas constant) to extract an estimate for the activation energy E_a, as shown in Figure 1c (main text). We note that we can also fit the PL rise curves using two exponential functions corresponding to a short and long time scale[14,15], but Arrhenius fits for each component separately yield similar activation energies. It is likely that the curves follow much more complicated functions and will need a more detailed analysis, but we simply use exponential functions to give estimates for the time scales involved.

Supplementary Note 7. Excitation Intensity-Dependence of Photoluminescence Changes

We present the results for different excitation intensities in Supplementary Fig. 7, where we observe that the trap densities and the time taken to reach stabilized emission varies dramatically with intensity, but ultimately similar total photon dose to reach stabilization for each case (e.g. ~200-300 J cm^{-2} at room temperature). At low excitation fluences, corresponding to photo-excited densities of ~$10^{15}cm^{-3}$ per pulse (1 MHz repetition rate), stabilized emission is reached only at times >10,000 s (~3 hours). This compares to thousands of seconds (~10 minutes) at the intermediate fluences (~$10^{16}cm^{-3}$ per pulse, 1 MHz repetition rate) also shown in Figure 1a of the main text, and only ~20 seconds for the highest fluences (~$10^{17}cm^{-3}$ per pulse, 1 MHz repetition rate). Consistent with previous reports[10,16,17], we see a transition at an excitation density of

~10^{17}cm^{-3} from the trap-limited monomolecular kinetics (as seen in Figure 1a) to bimolecular-dominating kinetics, in which the traps are predominantly filled.

Supplementary Note 8. Relation Between Trap Filling and Rise Times

We have previously reported steady state photoconductivity values of 10^{-3}–10^{-2} S cm^{-1} at excitation fluences approximately similar to solar insolation (~10^{17} cm^{-2} s^{-1})[18]. Assuming a carrier mobility of ~1-10 cm^{2} V^{-1} s^{-1},[18-20] this requires charge densities of ~10^{15}-10^{17} cm^{-3}. To obtain such charge densities at fluences of ~10^{17} cm^{-2} s^{-1} (~10^{21} cm^{-3} s^{-1}), carrier lifetimes of 10-100 μs are required. Since it is commonly accepted that one of the free carriers (likely the electron) is trapped within 100 ns - 1 μs[10,18,21,22], this long lifetime must be that of the residual (non-trapped) carriers and is likely an underestimate because not all carriers will be trapped (some recombine radiatively). By solving simplified rate equations for the two processes of trap filling (100-ns time scale with typical trap densities of ~10^{16}cm^{-3} [10]) and trap depopulation (100 μs as a conservatively slow estimate[10]), it will not take longer than a few 100s of microseconds for the system to reach equilibrium. This implies that the slow (minutes) transient phenomena observed in this work are not primarily attributed to simple trap filling effects.

Supplementary Note 9. Scanning Electron Microscopy (SEM) Grain Analysis

We show a grain size analysis via scanning electron microscopy (SEM) in Supplementary Fig. 8.

Supplementary Note 10. Local Photo-induced Cleaning

We show how the PL enhancement closely follows the laser profile in Supplementary Fig. 9.

Supplementary Note 11. Film Excitation and Relaxation in the Dark

Supplementary Fig. 11 shows the changes of the bulk PL intensity after varying lengths of time in the dark. Here, the films are illuminated (spot size waist w ~ 17 μm) until they reach a stabilized emission, then the laser is switched off for a fixed length of time and then switched back on, and the PL (at the same spot) continually monitored. Supplementary Fig. 11a shows the situation where the PL is reducing over time in the dark, where the inset shows the PL recovery value as a function of time left in the dark. This suggests that the photo-induced changes can be at least in part reversible and the films can eventually recover to lower stabilized emission levels over a time scale of hours, which is consistent with the microscale PL measurements (Supplementary Fig. 10). In contrast, Supplementary Fig. 11b shows a situation in an identical film measured under identical conditions, and in this case the PL continues increasing over time in the dark although eventually does seem to decrease over very long time scales. These results suggest that the changes that the illumination triggers can continue even while the film is kept in the dark. More generally, we see that the PL moves through long-term 'phases', i.e. over very long time scales (1000s of seconds), we see periods where the emission rises and other periods where it decreases even for the same film and spot. The rises or drops we see in the emission over time in the dark tend to follow the phase of the long term transient. This suggests that there are changes in the film that are instigated by illumination that are partly reversible but that will continue even without illumination. Similar long-term phenomena (long-term phases and associated rises or drops over time) were also reported in these perovskites by Gottesman et al. from photo-conductivity measurements[23], and they are also reminiscent of the photocurrent behavior under different bias conditions in solar cells[24]. We emphasize that here we are studying neat films with no contacts or applied bias. We

also note that we cannot exclude the possible contributions of atmospheric effects such as adsorbed oxygen or water species that remain in films even kept under vacuum for long periods of time[1-3].

We note that the photon dose used for collecting a fluorescence image (7 J cm^{-2}) is only ~2% of the photon dose the film was exposed to under simulated sunlight (360 J cm^{-2}) suggesting that the changes observed in Figure 3 (main text) are induced by the long light soak and not by the laser excitation required to collect a fluorescence image. This idea is further supported by the negligible changes in PL observed for a control film that had not been exposed to simulated AM 1.5 sunlight (Supplementary Fig. 12).

Supplementary Note 12. Time-of-flight Second Ion Mass Spectrometry (ToF-SIMS)

For the ToF-SIMS measurements, we first light soak the spot to be analyzed (indicated by the red circle in Supplementary Fig. 13a) and then immediately transfer the film in the dark to the ToF-SIMS instrument and put the sample under ultra-high vacuum (~ 40 minutes). Instrument calibration takes an additional 20 minutes, so in total the film is in the dark for ~ 60 minutes before being depth profiled. We believe the local changes in PL are still retained based on the results reported in Supplementary Figs. 11 and 12 (~9 hrs until stabilization) and therefore any changes in composition should also be retained. ToF-SIMS is a surface-sensitive technique with typical molecular ion escape depths of a few nanometers[25]. For depth profiling, the signal intensity is proportional to the composition at the top surface of the film after each sputter cycle. We identified and analyzed several different negative ion fragments and report the depth-summed ToF-SIMS images most representative of the data set (Supplementary Fig. 13), including I^-, I_2^-, PbI_2^-, PbI_3^-, $Pb_2I_5^-$, $Pb_3I_7^-$, PO_3^-, and $C_3HN_2^-$ (a fragment of methylammonium). In Supplementary Fig. 13a, we

show the iodide counts summed through the film depth and also define regions of interest for the illuminated region (red circle), an adjacent region (green circle) and a background region far from illumination (blue circle). As ToF-SIMS is primarily a qualitative technique, obtaining absolute changes in iodine content is not possible without careful calibration, and is therefore beyond the scope of this work. In order to extract an approximate relative change in intensity of iodine-containing fragments (R) between light-soaked, adjacent, and background regions, we used the following equation:

$$R = \sum_{i}^{N} \left(\frac{M_i^-(ROI) - M_i^-(Backgrd)}{M_i^-(Backgrd)} \right) \times 100\% \quad (13),$$

where $M_i^-(\dots)$ denotes the intensity of the iodine-containing fragment in the region of interest. To a first-order approximation, we estimate R= -1.2% in the illuminated region and R= +1.4% in the adjacent region, indicating that iodine has been partially redistributed. These values are significant given we only observe ~0.5% variations in several background regions, though we emphasize again here that there is likely a large error without proper calibration.

We briefly consider whether illumination could induce local variations in sputtering rates and ion extraction leading to artifacts in the intensity maps. In this possible scenario, we would expect the adjacent region (red circle in Supplementary Fig. 13a) to have a similar depth profile and intensity as the background region (blue circle in Supplementary Fig. 13a) – both of which have not been illuminated. In contrast, we still observe distinct profiles and intensities in the adjacent and background regions (Supplementary Fig. 14). This strongly suggests that material is moving laterally outside the illumination region. In addition, there are no other high-yield iodine-containing fragments with similar intensity maps as iodide. If illumination was changing the

sputtering rate and probability of ion extraction, we would expect a systematic artifact in at least some of the other fragment intensity maps.

Supplementary Note 13. Energy-Dispersive X-Ray Spectroscopy (EDS)

We show a semitransparent scanning electron microscope (SEM) image of the film in Supplementary Figure 16a overlaid on a PL map, again highlighting bright and dark spots. We show energy dispersive X-Ray spectroscopy (EDS) measurements at a dark (Supplementary Fig. 16b) and a bright (Supplementary Fig. 16c) spot to monitor the weight fractions of lead and iodide. Here, we use a high energy 10 keV electron beam to generate electron-hole pairs via inelastic scattering within the material; this same mechanism is exploited in order to detect electron beam-induced current (EBIC) measurements in photovoltaic devices[26,27] and cathodoluminescence measurements[28]. We estimate a peak electron-hole generation rate of $2x10^{23}$ $cm^{-3}s^{-1}$ (see below), hence we are probing the samples under carrier densities similar to the PL measurements and ~10 sun solar illumination conditions (~$3x10^{23}$ $cm^{-3}s^{-1}$). We note that carriers generated from the electron beam are distributed further in the bulk compared to carriers generated by photoexcitation (Supplementary Fig. 16d). We find that for the dark spot, which is associated with a rise in PL over time under illumination, the iodide content reduces on a time scale consistent with the rise times in PL and device open-circuit voltage at 1-sun equivalent optical irradiation (Figure 1b inset, main text)[10]. In contrast, the bright spot has very little change in iodide content over time under electron excitation. As a reference point, the lead content remains essentially unchanged in both cases. We note that the weight fraction values are dependent on the local interaction volume and include all detected elements including those in the substrate, and therefore the absolute weight fractions should not be compared. We also cannot rule out local differences in volatization of

iodide-containing species under high-energy electron excitation. Nevertheless, these EDS results suggest that the dark regions with high trap densities correspond to regions with excess mobile iodide, in agreement with the ToF-SIMS measurements.

We estimate that the pulsed excitation for PL measurements (~1 MHz, 0.5 μJ cm^{-2} per pulse), gives a peak charge density at t=0 of ~10^{16} cm^{-3} and an average density over the decay of ~10^{15} cm^{-3}. This is roughly equivalent to the charge density arising from 1-sun equivalent irradiation (upper bound charge density of ~10^{15} cm^{-3})[10,12,17]. We approximate the electron-hole generation rate under optical excitation using the average power output of the laser and by using the absorption coefficient reported elsewhere[29], and we plot the resulting profile in Supplementary Fig. 16d (black circles). We estimate the electron beam generation rate as a function of depth using established cathodoluminescence equations[30] taking into consideration the measured beam current (53.5 pA), perovskite density (4.286 g cm^{-3})[31], and perovskite bandgap (1.55 eV)[32]; we also plot this in Supplementary Fig. 16d (green squares).

The maximum generation rate is quite similar for both photon and electron-beam excitations. For the PL experiments, we obtain a maximum rate of ~$3x10^{23}$ $cm^{-3}s^{-1}$ compared to ~$2x10^{23}$ $cm^{-3}s^{-1}$ for the electron beam measurements. However, the spatial generation profiles are quite different. The optical excitation generation rate peaks at the surface and drops off exponentially through the film following the Beer Lambert Law, while the electron beam generates most of the carriers deeper in the sample as a result of inelastic scattering.

Supplementary Methods

Open-Circuit Voltage Rises. Solar cells were fabricated on FTO-coated glass (Pilkington, 7 Ω sq^{-1}). Initially, FTO was removed from regions under the anode contact by etching the FTO with 2 M HCl and zinc powder. Substrates were then cleaned as for the microscope slides. A hole-blocking layer of compact TiO_2 was deposited by spin-coating a mildly acidic solution of titanium isopropoxide in ethanol, and annealed at 500°C for 30 min. The perovskite precursor solution was spin-coated and the substrates annealed as for the microscope slides. After cooling, the spiro-OMeTAD hole-transporting layer was then deposited from a 66-mM chlorobenzene solution containing additives of lithium bis(trifluoromethanesulfonyl)imide and 4-tert-butylpyridine. Finally, 120-nm-gold electrodes were thermally evaporated under vacuum of $\sim10^{-6}$ Torr, at a rate of ~0.1 nm s^{-1}, to complete the devices.

For the transient open-circuit voltage rises, the devices were illuminated with a continuous-wave laser source at a wavelength of 532 nm and an intensity of 60 mW cm^{-2}, giving an approximately equivalent photoexcitation density to the 100 $mWcm^{-2}$ AM 1.5 spectrum. The data were acquired using a sourcemeter (Keithley 2400, USA) coupled to a customized Labview program.

Preparation of Single Crystal $CH_3NH_3PbI_3$

Lead iodide (98%) and anhydrous γ-butyrolactone (> 99%) were purchased from Sigma Aldrich and used without further purification. Methylammonium iodide (MAI) was synthesized by reacting methylamine (33 wt% in EtOH, Sigma) with equimolar hydriodic acid (57 wt%, Sigma) in an ice bath. The reaction mixture was stirred for 60 minutes and the liquid was removed with a rotary evaporator. The crude MAI solid was redissolved with EtOH (> 99.5%, Sigma), then precipitated and washed with diethyl ether (> 99%, Sigma). The MAI was dried and transferred to

a nitrogen atmosphere. Our procedure for single crystal growth was based off of the inverse temperature crystallization (ITC) method of Saidaminov et al[6]. A 1.2 M solution of both PbI_2 and MAI (1:1 molar ratio) was prepared in anhydrous γ-butyrolactone. The solution, exposed to ambient conditions, was preheated to 60 °C. Approximately 2 mL of this solution was filtered using a 0.2 μm PTFE filter. The filtrate was added to a 4 mL vial which was sealed shut before submerging in an oil bath heated to 80 °C. Over the course of two days, the temperature of the oil bath was gradually increased to 110 °C. After the two days, a ~1 cm^3 $CH_3NH_3PbI_3$ single crystal was removed from the growth solution and washed with two 2 mL aliquots of acetophenone. The crystal was then dried and transferred into a N_2 filled glovebox. The crystal was cleaved in the glovebox using a razor blade and the PL was measured under nitrogen flow.

Preparation of $CH_3NH_3PbI_3$ ($PbCl_2$ precursor)

Thin films of $CH_3NH_3PbI_{3-x}Cl_x$ ($PbCl_2$ method) were formed by first preparing a 40% weight precursor solution consisting of 2.64 M MAI (Lumtec) and 0.88 M $PbCl_2$ (Sigma-Aldrich 99.99% purity) dissolved in DMF[4,5]. The solutions were spin-coated onto oxygen-plasma-etched glass at 2500 rpm for 60 s in a nitrogen filled glovebox and the substrates subsequently dried at room temperature for 20 mins and then annealed at 90°C for 2 hours.

Preparation of $CH_3NH_3PbI_3$ (Dripping Method)

Thin films of $CH_3NH_3PbI_3$ were also prepared using the solvent engineering ('dripping') method described in detail elsewhere[33,34]. In brief, MAI was synthesized and purified as described above. Equimolar (0.75 M) solutions of MAI and PbI_2 (Alfa Aesar, 99.9985% purity) were prepared in DMF and spin-coated onto oxygen-plasma-etched glass at 5000 rpm for 35 s. After 6 s, 150 μL

chlorobenzene was deposited on the spinning sample in order to induce rapid crystallization. After spinning, the samples were immediately heated at 100 °C for 10 minutes.

X-Ray Diffraction (XRD) Measurements. X-ray diffraction (XRD) patterns of the films deposited on boron-doped Si substrates were collected using a Bruker D8 diffractometer equipped with a Cu Kα radiation source and operated at 40 mV and 40 mA. The patterns were collected while illuminating in-situ with a 532-nm CW laser. Further details, including photon doses, are included in the figure caption (Supplementary Fig. 3).

Energy-Dispersive X-ray Spectroscopy (EDS). EDS compositional data was taken on a FEI Sirion SEM using a 10 kV accelerating voltage on samples with approximately 7 nm of Au/Pd sputtered using a SPI-Module Sputter Coater with argon flow. The data was analyzed using AZtecEnergy EDS software package (Ver. 2.1). The EDS compositional dynamics were monitored using a video recording device; wt% values were updated by the AZtec software every 500 ms.

Supplementary References

1 Tian, Y. *et al.* Mechanistic insights into perovskite photoluminescence enhancement: light curing with oxygen can boost yield thousandfold. *Phys Chem Chem Phys* **17**, 24978-24987, doi:10.1039/c5cp04410c (2015).
2 Galisteo-López, J. F., Anaya, M., Calvo, M. E. & Míguez, H. Environmental Effects on the Photophysics of Organic–Inorganic Halide Perovskites. *J. Phys. Chem. Lett.* **6**, 2200-2205, doi:10.1021/acs.jpclett.5b00785 (2015).
3 Eperon, G. E. *et al.* The Importance of Moisture in Hybrid Lead Halide Perovskite Thin Film Fabrication. *ACS Nano* **9**, 9380-9393, doi:10.1021/acsnano.5b03626 (2015).
4 Eperon, G. E., Burlakov, V. M., Docampo, P., Goriely, A. & Snaith, H. J. Morphological Control for High Performance, Solution-Processed Planar Heterojunction Perovskite Solar Cells. *Advanced Functional Materials* **24**, 151-157, doi:10.1002/adfm.201302090 (2014).

5 Stranks, S. D. *et al.* Electron-hole diffusion lengths exceeding 1 micrometer in an organometal trihalide perovskite absorber. *Science* **342**, 341-344, doi:10.1126/science.1243982 (2013).

6 Saidaminov, M. I. *et al.* High-quality bulk hybrid perovskite single crystals within minutes by inverse temperature crystallization. *Nat Commun* **6**, doi:10.1038/ncomms8586 (2015).

7 Zhang, W. *et al.* Enhanced optoelectronic quality of perovskite thin films with hypophosphorous acid for planar heterojunction solar cells. *Nat Commun* **6**, 10030, doi:10.1038/ncomms10030 (2015).

8 Pathak, S. *et al.* Atmospheric influence upon crystallization and electronic disorder and its impact on the photophysical properties of organic-inorganic perovskite solar cells. *ACS Nano* **9**, 2311-2320, doi:10.1021/nn506465n (2015).

9 Zhou, H. *et al.* Interface Engineering of Highly Efficient Perovskite Solar Cells. *Science* **345**, 542-546, doi:10.1126/science.1254050 (2014).

10 Stranks, S. D. *et al.* Recombination Kinetics in Organic-Inorganic Perovskites: Excitons, Free Charge, and Subgap States. *Phys Rev Appl* **2**, 034007, doi:10.1103/Physrevapplied.2.034007 (2014).

11 Miyata, A. *et al.* Direct measurement of the exciton binding energy and effective masses for charge carriers in organic–inorganic tri-halide perovskites. *Nature Physics* **11**, 582-587, doi:10.1038/nphys3357 (2015).

12 D'Innocenzo, V. *et al.* Excitons versus free charges in organo-lead tri-halide perovskites. *Nat. Commun.* **5**, 3586, doi:10.1038/ncomms4586 (2014).

13 Wehrenfennig, C., Liu, M. Z., Snaith, H. J., Johnston, M. B. & Herz, L. M. Charge carrier recombination channels in the low-temperature phase of organic-inorganic lead halide perovskite thin films. *Apl Mater* **2**, 081513, doi:10.1063/1.4891595 (2014).

14 Hoke, E. T. *et al.* Reversible photo-induced trap formation in mixed-halide hybrid perovskites for photovoltaics. *Chem. Sci.* **6**, 613-617, doi:10.1039/c4sc03141e (2015).

15 Eames, C. *et al.* Ionic transport in hybrid lead iodide perovskite solar cells. *Nat Commun* **6**, 7497, doi:10.1038/ncomms8497 (2015).

16 Yamada, Y., Nakamura, T., Endo, M., Wakamiya, A. & Kanemitsu, Y. Photocarrier recombination dynamics in perovskite CH3NH3PbI3 for solar cell applications. *J Am Chem Soc* **136**, 11610-11613, doi:10.1021/ja506624n (2014).

17 deQuilettes, D. W. *et al.* Impact of microstructure on local carrier lifetime in perovskite solar cells. *Science* **348**, 683-686, doi:10.1126/science.aaa5333 (2015).

18 Leijtens, T. *et al.* Electronic properties of meso-superstructured and planar organometal halide perovskite films: charge trapping, photodoping, and carrier mobility. *ACS Nano* **8**, 7147-7155, doi:10.1021/nn502115k (2014).

19 Wehrenfennig, C., Eperon, G. E., Johnston, M. B., Snaith, H. J. & Herz, L. M. High Charge Carrier Mobilities and Lifetimes in Organolead Trihalide Perovskites. *Adv. Mater.* **26**, 1584-1589, doi:10.1002/adma.201305172 (2014).

20 Hutter, E. M., Eperon, G. E., Stranks, S. D. & Savenije, T. J. Charge Carriers in Planar and Meso-Structured Organic-Inorganic Perovskites: Mobilities, Lifetimes, and Concentrations of Trap States. *J. Phys. Chem. Lett.* **6**, 3082-3090, doi:10.1021/acs.jpclett.5b01361 (2015).

21 Wetzelaer, G. J. *et al.* Trap-assisted non-radiative recombination in organic-inorganic perovskite solar cells. *Adv Mater* **27**, 1837-1841, doi:10.1002/adma.201405372 (2015).

22 D'Innocenzo, V., Srimath Kandada, A. R., De Bastiani, M., Gandini, M. & Petrozza, A. Tuning the Light Emission Properties by Band Gap Engineering in Hybrid Lead Halide Perovskite. *J. Am. Chem. Soc.*, doi:10.1021/ja511198f (2014).
23 Gottesman, R. *et al.* Extremely Slow Photoconductivity Response of CH3NH3PbI3 Perovskites Suggesting Structural Changes under Working Conditions. *J. Phys. Chem. Lett.* **5**, 2662-2669, doi:10.1021/Jz501373f (2014).
24 Kim, H. S. & Park, N. G. Parameters Affecting I-V Hysteresis of CH3NH3PbI3 Perovskite Solar Cells: Effects of Perovskite Crystal Size and Mesoporous TiO2 Layer. *J. Phys. Chem. Lett.* **5**, 2927-2934, doi:10.1021/Jz501392m (2014).
25 Muramoto, S., Brison, J. & Castner, D. G. Exploring the Surface Sensitivity of TOF-Secondary Ion Mass Spectrometry by Measuring the Implantation and Sampling Depths of Bin and C60 Ions in Organic Films. *Analytical Chemistry* **84**, 365-372, doi:10.1021/ac202713k (2012).
26 Edri, E. *et al.* Why lead methylammonium tri-iodide perovskite-based solar cells require a mesoporous electron transporting scaffold (but not necessarily a hole conductor). *Nano Lett* **14**, 1000-1004, doi:10.1021/nl404454h (2014).
27 Edri, E. *et al.* Elucidating the charge carrier separation and working mechanism of CH3NH3PbI(3-x)Cl(x) perovskite solar cells. *Nat Commun* **5**, 3461, doi:10.1038/ncomms4461 (2014).
28 Bischak, C. G., Sanehira, E. M., Precht, J. T., Luther, J. M. & Ginsberg, N. S. Heterogeneous Charge Carrier Dynamics in Organic-Inorganic Hybrid Materials: Nanoscale Lateral and Depth-Dependent Variation of Recombination Rates in Methylammonium Lead Halide Perovskite Thin Films. *Nano Lett* **15**, 4799-4807, doi:10.1021/acs.nanolett.5b01917 (2015).
29 Green, M. A., Ho-Baillie, A. & Snaith, H. J. The Emergence of Perovskite Solar Cells. *Nature Photonics* **8**, 506-514, doi:10.1038/nphoton.2014.134 (2014).
30 Bonard, J. M., Ganière, J. D., Akamatsu, B., Araújo, D. & Reinhart, F. K. Cathodoluminescence study of the spatial distribution of electron-hole pairs generated by an electron beam in Al0.4Ga0.6As. *J. Appl. Phys.* **79**, 8693-8703, doi:10.1063/1.362560 (1996).
31 Baikie, T. *et al.* Synthesis and crystal chemistry of the hybrid perovskite (CH3NH3) PbI3 for solid-state sensitised solar cell applications. *J. Mater. Chem. A* **1**, 5628-5641, doi:Doi 10.1039/C3ta10518k (2013).
32 Eperon, G. E. *et al.* Formamidinium lead trihalide: a broadly tunable perovskite for efficient planar heterojunction solar cells. *Energy Environ. Sci.* **7**, 982-988, doi:10.1039/c3ee43822h (2014).
33 Xiao, M. *et al.* A fast deposition-crystallization procedure for highly efficient lead iodide perovskite thin-film solar cells. *Angew Chem Int Ed* **53**, 9898-9903, doi:10.1002/anie.201405334 (2014).
34 Jeon, N. J. *et al.* Solvent Engineering for High-Performance Inorganic-Organic Hybrid Perovskite Solar Cells. *Nat Mater* **13**, 897-903, doi:10.1038/nmat4014 (2014).
